# Changes in Physical Activity After Installation of a Fitness Zone in a Community Park

**DOI:** 10.5888/pcd15.170560

**Published:** 2018-08-09

**Authors:** Mojgan Sami, Megan Smith, Oladele A. Ogunseitan

**Affiliations:** 1 Department of Population Health and Disease Prevention, Program in Public Health, University of California, Irvine; 2 Department of Statistics, University of California, Irvine; 3 School of Social Ecology, University of California, Irvine

## Abstract

**Introduction:**

Increases in physical activity can lead to decreases in the prevalence of chronic diseases. Parks provide an ideal setting for physical activity. We investigated the effect of a fitness equipment installation on the intensity of park users’ physical activity at a community park.

**Methods:**

We used the System for Observing Play and Recreation in a Community to record physical activity in Eastgate Park in Garden Grove, California, in August 2015 (preintervention [n = 1,650 person-periods]) and in February 2016 (postintervention [n = 1,776 person-periods]). We quantified physical activity in target areas of the park during 15-minute observation periods in 2 ways: 1) we categorized each user’s activity level during the period (sedentary, walking, vigorous), and 2) we converted activity levels to numeric metabolic equivalent task (MET) scores and calculated the period-average score across users. We used mixed-effects regression models to assess 1) the proportional odds of higher activity level at postintervention and 2) the association between intervention status (pre vs post) and mean period-average MET scores.

**Results:**

In the immediate zone around the fitness equipment, the odds ratio for a higher activity level was 1.58 (95% confidence interval [CI], 1.14–2.18; *P* = .006) and the mean period-average MET score was 0.33 (95% CI, −0.07 to 0.74; *P* = .11) units higher at postintervention. Across the park as a whole, the odds ratio for a higher activity level was 1.41 (95% CI, 1.21–1.63; *P* < .001), and the mean period-average MET score was 0.34 (95% CI, 0.12–0.56; *P* = .003) units higher at postintervention.

**Conclusion:**

Installing fitness zones appears to be an effective intervention for increasing physical activity of park users. Further studies need to be conducted to understand the sustained impact of fitness zones over time.

## Introduction

Physical inactivity contributes to obesity and chronic diseases such as cardiovascular impairment and diabetes (1). However, less than 5% of American adults meet the national moderate-to-vigorous physical activity target of 150 minutes per week (2). Strategies to increase physical activity are a recognized pathway to decreasing the prevalence of preventable chronic diseases (3). Public health and urban planning link the built environment to the promotion or deterrence of physical activity (2,4,5). Parks provide an ideal setting for physical activity. Public health agencies advocate community-based environmental interventions, such as those implemented in parks, to improve access to physical activity facilities (2,5,6), and health professionals prescribe park visits (7,8). Cities are improving parks, and these improvements can lead to increases in physical activity levels (9,10). In 2016, the city of Garden Grove, California, installed outdoor exercise equipment, or a fitness zone (11), in its Eastgate Park.

Several studies show that fitness zones are associated with increases in physical activity (10,12), while other studies show no significant correlation (13). Our study investigated the effect of the installation of fitness equipment on physical activity in Eastgate Park. The primary objective of our study was to describe the distribution of park users’ observed activity levels (sedentary, walking, and vigorous activities) before and after installation of the fitness equipment and to quantify the association between intervention status (preintervention vs postintervention) and activity level. The secondary objective was to investigate the association between the intervention and the period-average metabolic equivalent task (MET) score, a composite score described elsewhere (14). The period-average MET score represents the average physical activity intensity during an observation period. Our findings will help us better understand the effectiveness of fitness zones as a strategy to increase physical activity with the goal of reducing the prevalence of preventable chronic diseases.

## Methods

**Figure 1:**
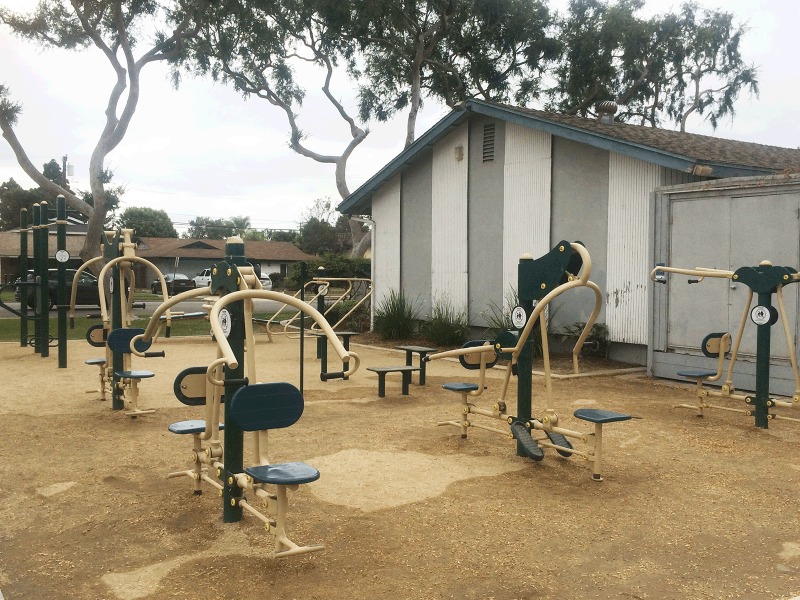
Fitness zone in Eastgate Park, Garden Grove, 2016. Photography by Mojgan Sami.

Eastgate Park is a 4.5-acre park in a suburban community in northern Orange County and has amenities such as open green space, a children’s playground, a community pool, a meeting facility, and a covered picnic area. The fitness zone in Eastgate Park was installed in December 2015 in a previously open space. The city’s parks and recreation department worked with Greenfields Outdoor Fitness Equipment, Inc (15), an Orange County–based company, to install 8 pieces of equipment: a 2-person lateral pull, a 2-person back and arms combination resistance, a 2-person vertical arm press, a 2-person chest press, a set of combination bars, a 2-person sit-up bench, a parallel dip and stretch machine, and polymetric steps (Figure 1). 

**Figure 2:**
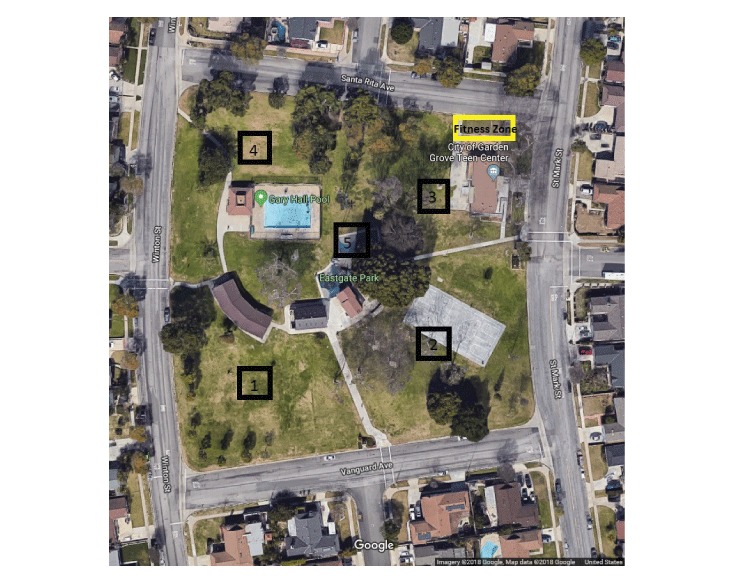
Aerial map of Eastgate Park, Garden Grove, 2015. Researchers divided the park into 5 target areas. Target area 3 encompasses the site of the fitness zone, which was installed in December 2015. Image obtained from Google Maps (www.google.com/maps/place/Eastgate+Park/@33.7884134,-118.0346572,156m/data=!3m1!1e3!4m5!3m4!1s0x80dd2f26e4a34b8b:0xfb28efa20564369!8m2!3d33.788409!4d-118.03411), and graphics were added by the researchers.

The institutional review board of the University of California, Irvine, deemed that this observational study in a public park required no review because it involved no direct interaction with human participants. To assess park users’ levels of physical activity preintervention (before installation of equipment) and postintervention (after installation), we used the System of Observing Play and Recreation in Communities (SOPARC), a standardized observation tool (16). To quantify physical activity in the park as a whole, we divided Eastgate Park into 5 target areas (Figure 2). Trained researchers made preintervention observations during 3 days in 2015 (August 29, August 31, and September 2) and postintervention observations during 3 days in 2016 (February 1, February 3, and February 6). Each target area was monitored by a researcher during 4 one-hour intervals (7:00 to 8:00 AM, 12:00 to 1:00 PM, 3:30 to 4:30 PM, and 6:00 to 7:00 PM) on all 3 study days (Monday, Wednesday, and Saturday). According to SOPARC guidelines, we divided each hour into 15-minute increments, and we sampled user activity continuously during each increment.

One researcher observed each assigned target area for the duration of the 1-hour interval, per SOPARC protocol. The researcher recorded the sex, age group, and race/ethnicity of each user and categorized each user’s activity level as sedentary, walking, or vigorous. Researchers coded equipment use in the fitness zone as sedentary when the machines were not used as intended (eg, used as seating rather than exercise); they coded activity as vigorous when each apparatus was used as instructed. The smallest unit of measurement in the SOPARC data is 1 person-period, defined as the contribution to the study of a single park user who occupied a target area for all or part of a 15-minute period. If a park user occupied the target area longer than a single 15-minute period, or if a park user left the target area and returned during a later period, researchers created multiple unique person-period records. We calculated 1,650 person-periods at preintervention and 1,776 person-periods at postintervention. 

The activity levels for park users were subsequently converted into MET scores. There are only 3 possible MET scores: sedentary is equal to 1.5 METs, walking is equal to 3.0 METs, and vigorous is equal to 6.0 METs (14). We calculated a period-average MET score for each observation period in a target area by averaging the individual MET scores for all person-periods in the period. The period-average MET scores quantify the overall average level of user activity in each target area during each observation period.

We used χ^2^ tests to examine the demographic distribution of park users preintervention and postintervention.

### Proportional odds mixed-effects regression model

We used a proportional odds mixed-effects regression model to estimate the odds ratios for a higher activity level at postintervention than at preintervention. The dependent variable, activity level, is ordered, with vigorous being the highest level, followed by walking and then sedentary. The unit of analysis in these models is the person-period. The proportional odds model was adjusted for age group, sex, race/ethnicity group, and day of week (weekday vs weekend day). To account for correlation among observations measured at the same time of day or in the same target area, random intercepts for time and target area were included. The model has the following form:


*L*
^C^
*_ijk_* = β_0_ + β_P_
*X_ijk_* + β*_D_D_ijk_* + β*_A_A_ijk_* + β*_S_S_ijk_* + β*_E_E_ijk_* + *a_j_* + *b_k_* + ε*_ijk_*

Here, the index *i* indicates an individual person-period, *j* indicates time of day, and *k* indicates target area. The log odds of a park user’s activity being classified above category *C* versus into or below *C* (for category *C* = walking) or versus into C (for category *C* = sedentary) is denoted by *L^C^_ijk_*. The variable *X_ijk_* is intervention status (post = 1, pre = 0), *D_ijk_* indicates day of week, *A_ijk_* is the age group, *S_ijk_* is sex, and *E_ijk_* is the race/ethnicity corresponding to the park user. The overall intercept for the proportional odds model is β_0_, and β*_P_* is the coefficient associated with intervention status. The random intercepts are denoted by *a_j_* (time of day) and *b_k_* (target area); ε*_ijk_* denotes the random within-person-period error and is assumed to be an independent normally distributed random variable. Similar proportional odds models stratified by target area were also fit to examine the association between activity level and intervention status (preintervention vs postintervention) in each target area. Some target areas had distinct built environment elements or patterns of usage. The stratified models were designed to determine in which of the 5 target areas, if any, changes in physical activity postintervention occurred and which areas of the park were the strongest drivers of the estimated changes in activity for Eastgate Park overall. We tabulated odds ratios (ORs) and 95% confidence intervals (CIs).

### Linear mixed-effects regression model

To assess the association between period-average MET score and park intervention status, we fit a linear mixed-effects regression model with period-average MET score as the outcome and period as the unit of analysis. The model was adjusted for day of week (weekday vs weekend day). Random intercepts for time of day and target area were included to account for correlation among measurements taken during the same time of day or within the same target area. The model has the following form:

Y*_ijk_* = β_0_ + β_P_
*X_ijk_* + β*_D_D_ijk_* + *a_j_ + b_k_* + ε*_ijk_*

Here, the index *i* catalogs individual periods, *j* indicates time of day, and *k* denotes target area. The outcome *Y_ijk_* is the period-average MET score, and the variable *X_ijk_* is intervention status (post = 1, pre = 0) for the period. The overall intercept for the linear model is β_0_, and β*_P_* is the coefficient associated with intervention status. The random intercepts are denoted by *a_j_* (time of day) and *b_k_* (target area). The random within-period error in the period-average MET score, denoted ε*_ijk_*, is assumed to be an independent normally distributed random variable. We subsequently conducted a stratified analysis of the association between period-average MET and intervention status within each target area. The resulting regression coefficients for intervention status yielded the estimated differences in mean period-average MET score (either overall or by target area) comparing postintervention park use with preintervention park use.

## Results

**Figure 3:**
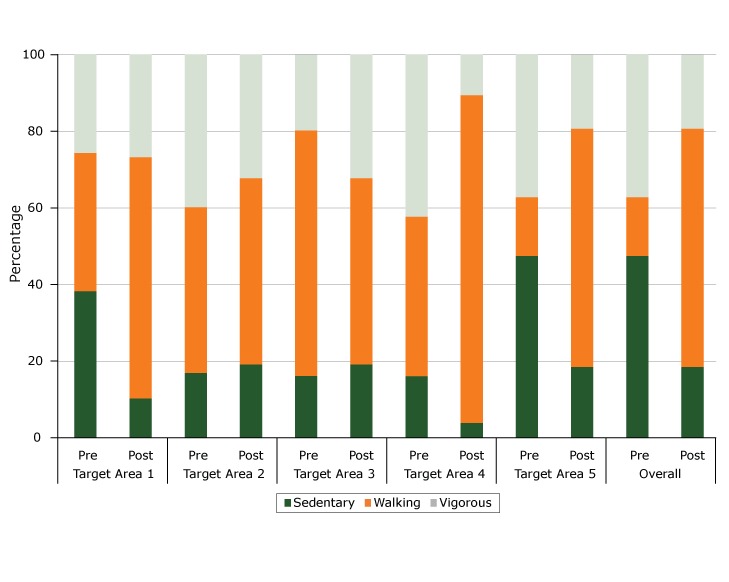
Distribution of activity levels in Eastgate Park at preintervention and postintervention, by target area and overall, Garden Grove, California, 2015–2016. Percentages were calculated on the basis of person-periods, defined as the contribution to the study of a single park user who occupied a target area for all or part of a 15-minute period. **Table:** 

We found significant differences in the observed demographic distribution of park users before and after installation of the fitness zone (Table 1). The proportions of sedentary, walking, and vigorous activity levels observed among park users differed from preintervention to postintervention by target area and in the park overall (Figure 3). In target area 5, the distribution of activity levels for the person-period observations at preintervention (47.5% sedentary, 15.3% walking, 37.3% vigorous) was markedly different from the distribution of activity levels for person-period observations at postintervention (18.6% sedentary, 62.1% walking, 19.2% vigorous). Postintervention users in the park overall were estimated to have 41% higher odds of being classified in a more active category than were preintervention users with similar demographic characteristics (OR = 1.41; 95% CI, 1.21–1.63, *P* < .001) (Table 2). Among the 5 target areas, postintervention users had significantly higher odds of being observed at a higher activity level in target area 1 (OR = 2.11; 95% CI, 1.51–2.97; *P* < .001), target area 3, the location of the fitness zone (OR = 1.58; 95% CI, 1.14–2.18; *P* = .006), and target area 5 (OR = 1.97; 95% CI, 1.34–2.89; *P* < .001).

**Table 1:** Percentage Distribution of Demographic Characteristics Among Park Users, Overall and by Target Area^a^, Preintervention and Postintervention^b^, Eastgate Park, Garden Grove, California, 2015–2016

Characteristic	Target Area 1		Target Area 2		Target Area 3		Target Area 4		Target Area 5		Overall	
	Pre	Post	Pre	Post	Pre	Post	Pre	Post	Pre	Post	Pre	Post
**Total no. of person-periods^c^**	378	347	261	590	294	371	357	151	360	317	1,650	1,776
**Sex**												
% Male	50.0	71.2	57.1	64.2	51.4	62.3	48.2	58.9	41.4	54.9	49.1	63.1
% Female	50.0	28.8	42.9	35.8	48.6	37.7	51.8	41.1	58.6	45.1	50.9	36.9
χ^2^ (*P* value)^d^	33.0 (<.001)		3.6 (.06)		7.5 (.006)		4.5 (.03)		11.8 (<.001)		67.3 (<.001)	
**Age group**												
% Child	22.8	12.7	19.2	17.8	32.0	16.4	30.8	7.9	43.3	32.8	30.1	18.4
% Teen	9.5	22.8	20.7	30.2	19.4	29.9	10.6	11.3	5.6	17.0	12.4	24.7
% Adult	48.9	45.0	44.8	44.6	36.4	44.2	45.9	57.0	41.9	44.5	43.9	45.6
% Senior	18.8	19.6	15.3	7.5	12.2	9.4	12.6	23.8	9.2	5.7	13.6	11.3
χ^2^ (*P* value)^d^	30.9 (<.001)		17.5 (<.001)		27.8 (<.001)		34.2 (<.001)		28.2 (<.001)		121.9 (<.001)	
**Race/ethnicity**												
% White	65.9	61.4	64.8	52.9	58.8	69.5	57.1	74.2	52.5	59.0	59.6	60.9
% Hispanic	13.8	32.0	17.2	35.9	20.7	19.7	5.0	19.9	7.2	23.7	12.2	28.2
% Black	1.3	1.7	1.1	2.7	2.0	3.0	3.6	0	2.8	12.0	2.2	4.0
% Other	19.0	4.9	16.9	8.5	18.4	7.8	34.2	6.0	37.5	5.4	25.9	6.9
χ^2^ (*P* value)^d^	57.0 (<.001)		38.9 (<.001)		18.2 (<.001)		67.9 (<.001)		129.5 (<.001)		307.8 (<.001)	

a For the purposes of the study, researchers divided the 4.5-acre Eastgate Park into 5 target areas, one of which, target area 3, encompassed the fitness zone, which consisted of 8 pieces of newly installed fitness equipment.

b The fitness zone was installed in December 2015. Preintervention refers to data collection in August 2015, before installation, and postintervention refers to data collection in February 2016, after installation.

c Person-periods defined as the contribution to the study of a single park user who occupied a target area for all or part of a 15-minute period.

d Tests compare distribution of characteristics before and after installation of fitness zone; a type I error rate of .05 was used to calculate *P* values for these tests.

Target Area	Intervention Status	Percentage	Activity Level
1	Pre	38.3	Sedentary
1	Pre	36.1	Walking
1	Pre	25.7	Vigorous
1	Post	10.4	Sedentary
1	Post	62.8	Walking
1	Post	26.8	Vigorous
2	Pre	17.0	Sedentary
2	Pre	43.2	Walking
2	Pre	39.8	Vigorous
2	Post	19.3	Sedentary
2	Post	48.5	Walking
2	Post	32.2	Vigorous
3	Pre	16.3	Sedentary
3	Pre	63.9	Walking
3	Pre	19.7	Vigorous
3	Post	12.9	Sedentary
3	Post	56.3	Walking
3	Post	30.7	Vigorous
4	Pre	16.2	Sedentary
4	Pre	41.5	Walking
4	Pre	42.3	Vigorous
4	Post	4.0	Sedentary
4	Post	85.4	Walking
4	Post	10.6	Vigorous
5	Pre	47.5	Sedentary
5	Pre	15.3	Walking
5	Pre	37.3	Vigorous
5	Post	18.6	Sedentary
5	Post	62.1	Walking
5	Post	19.2	Vigorous
Overall	Pre	47.5	Sedentary
Overall	Pre	15.3	Walking
Overall	Pre	37.3	Vigorous
Overall	Post	18.6	Sedentary
Overall	Post	62.1	Walking
Overall	Post	19.2	Vigorous

**Table 2:** Proportional Odds Mixed-Effects Regression Model: Estimated Odds^a^ of Having a Higher Activity Level at Postintervention Than at Preintervention^b^, Overall and by Target Area^c^, Eastgate Park, Garden Grove, California, 2015–2016

Park Area	Activity-Level Odds Ratio^a^ (95% Confidence Interval)	*P* Value
Target area 1	2.11 (1.51–2.97)	<.001
Target area 2	0.91 (0.66–1.26)	.58
Target area 3	1.58 (1.14–2.18)	.006
Target area 4	0.80 (0.53–1.22)	.30
Target area 5	1.97 (1.34–2.89)	<.001
Overall	1.41 (1.21–1.63)	<.001

a The odds ratio for a higher activity level compares postintervention users to preintervention users of the same observed age group, sex, and racial/ethnic group, and who were observed on the same type of day (weekday or weekend day).

b The fitness zone was installed in December 2015. Preintervention refers to data collection in August 2015, before installation, and postintervention refers to data collection in February 2016, after installation.

c For the purposes of the study, researchers divided the 4.5-acre Eastgate Park into 5 target areas, one of which, target area 3, encompassed the fitness zone, which consisted of 8 pieces of newly installed fitness equipment.

The mean period-average MET score at preintervention was 3.20 (25th percentile, 2.50; 50th percentile, 3.32; 75th percentile, 4.00). At postintervention, the mean period-average MET score was 3.52 (25th percentile, 3.00; 50th percentile, 3.16; 75th percentile, 4.00). Controlling for day of week, the mean period-average MET score at postintervention in the park overall was an estimated 0.34 (95% CI, 0.12–0.56, *P* < .001) units higher than the mean period-average MET score at preintervention (Table 3). Among the 5 target areas, the mean period-average MET score was significantly higher at postintervention than at preintervention in target area 1 (0.57 units; 95% CI, 0.07–1.07; *P* = .03) and in target area 5 (0.69 units; 95% CI, 0.21–1.16; *P* = .005). In target area 3, the location of the fitness zone, the mean period-average MET score at postintervention was 0.33 (95% CI, −0.07 to 0.74; *P* = .11) units higher than at preintervention.

**Table 3:** Linear Mixed-Effects Regression Model: Estimated Difference in Mean Period-Average MET Scores^a^ Between Preintervention and Postintervention,^b^ Controlling for Day of Week (Weekday or Weekend), Overall and by Target Area^c^, Eastgate Park, Garden Grove, California, 2015–2016

Park Area	Estimated Difference (95% Confidence Interval)	*P* Value
Target area 1	0.57 (0.07 to 1.07)	.03
Target area 2	0.17 (−0.25 to 0.59)	.37
Target area 3	0.33 (−0.07 to 0.74)	.11
Target area 4	−0.21 (−0.76 to 0.34)	.45
Target area 5	0.69 (0.21 to 1.16)	.005
Overall	0.34 (0.12 to 0.56)	.003

Abbreviation: MET, metabolic equivalent task.

a The period-average MET score quantifies the overall average level of user activity in each target area during each observation period. A MET score of 1.5 is equal to sedentary; 3.0, walking; 6.0, vigorous.

b The fitness zone was installed in December 2015. Preintervention refers to data collection in August 2015, before installation, and postintervention refers to data collection in February 2016, after installation.

c For the purposes of the study, researchers divided the 4.5-acre Eastgate Park into 5 target areas, one of which, target area 3, encompassed the fitness zone, which consisted of 8 pieces of newly installed fitness equipment.

## Discussion

Our findings support the hypothesis that physical activity levels among Eastgate Park users would be higher after a public health intervention consisting of the installation of fitness equipment, where individuals’ physical activity is categorized as sedentary, walking, or vigorous through use of the SOPARC assessment tool. We found strong evidence of an increase in Eastgate Park as a whole, both when physical activity was measured at the level of the individual park user person-period and when physical activity was quantified by using the aggregate measure of the period-average MET score.

Furthermore, after installation of the fitness zone, we found significantly higher levels of physical activity in particular target areas of the park. Community park users had higher odds of being observed at a more intense activity level at postintervention than at preintervention in target area 1, target area 3 (location of the fitness equipment), and target area 5. The increase in moderate and vigorous activity after the installation of the fitness zone was not surprising. The intervention was associated with increased activity in target area 1 and target area 5 in terms of period-average MET scores as well.

Target area 1 is an open green space that researchers observed to be used as a shortcut through the park. No data were systematically collected to describe park users walking through the park (rather than using the park as a destination). To distinguish between passive and active use of parks, future studies should investigate how the location of a park and its proximity to residential neighborhoods and transit corridors affect park use. Target area 5 has a playground and a pool. The pool was closed in February, during postintervention data collection. The seasonally dependent physical activity in target area 5 illustrates one limitation of our study: preintervention and postintervention data collection periods were not matched on weather and temperature. In target area 5, the distribution of activity levels at preintervention was markedly different from the distribution at postintervention. This difference in distribution reflects seasonal differences in park use: stationary sunbathing and active pool play in August and generally walking past the closed pool area in February. The potential impact of the fitness zone could be partially obscured in our analysis because of these seasonal differences. When target area 5 was excluded from the overall analysis, the odds ratio for a higher activity level in the park overall was 1.33 (95% CI, 1.12–1.57, *P* = .001).

Research supports the connection between park space and walking activities (17). Indeed, we found walking to be the dominant activity observed in Eastgate Park at postintervention. Interestingly, walking activities increased in the park after the fitness zone installation, suggesting that park improvements may motivate activity because of their novelty. For example, people may have been walking in Eastgate Park to see the fitness equipment out of curiosity. Future studies could investigate the value of periodic changes to a park’s built environment to continually attract visitors and provide inspiration for exploration and activity.

Organized activities and outreach are pivotal to increasing a community’s usage of a neighborhood park, and community-based participation can contribute to increased physical activity levels (17,18). Our data show the immediate impact of the fitness zone in Eastgate Park. The fitness zone installation could be associated with even greater increases in physical activity after the community has more time to discover, engage with, and learn proper usage of the equipment. On the other hand, without continued community engagement, use of the fitness zone may decrease. Future studies could assess whether park use and physical activity in Eastgate Park increase after additional time and community engagement.

Our study has several limitations. Although our findings support a strong association between pre–post intervention status and observed physical activity, we cannot draw conclusions on causality without considering other potential explanations for the observed associations, such as season of year or calendar time, and other unmeasured changes in the Garden Grove community between preintervention and postintervention. Practical considerations related to grant guidelines, community partnerships, and academic scheduling required us to conduct preintervention and postintervention observations during 2 different seasons; future studies would ideally compare preintervention and postintervention physical activity in the park under similar environmental conditions. Comparisons of study data collected during different seasons in a temperate climate, such as southern California, however, are likely to be less problematic than comparisons of study data collected in a location with more extreme seasonal climate fluctuations. Other limitations include possible misclassification of the physical activity outcome variables or demographic descriptors and possible (conscious or unconscious) bias among the researchers collecting data, who were aware of the purpose and timing of the intervention and data collection. Although it is important to be cautious about generalizing this study’s results to other contexts, these findings do support park infrastructure improvements as a vehicle for health-promoting behavior in Eastgate Park.

Finally, although our study provides clear evidence of increased levels of physical activity after the installation of the fitness zone, the SOPARC measure does not account for contextual information that may explain this increase. We found significant differences in the observed demographic makeup of park users before after installation of the fitness zone; thus, it is important to control for sex, age group, and race/ethnicity in the analysis of park users’ physical activity in future studies. We recommend more qualitative and ethnographic studies to explore the increase in physical activity. Such studies may be of value in understanding the reasons for observed demographic differences in physical activity levels after the installation of fitness zones.

To promote physical activity as a strategy for preventing chronic diseases, it is beneficial to evaluate park design and sustain community outreach efforts to promote knowledge of park amenities. Characteristics such as accessibility, safety, quality of amenities, and park maintenance are important considerations for physical activity interventions. Park programming also attracts park users (19). The Centers for Disease Control and Prevention-coordinated Partnerships to Improve Community Health, which funded the interventions in Eastgate Park, provides an opportunity to generate and aggregate evidence nationwide about interventions that promote physical activity. Although we found overall physical activity to increase in the park after the fitness zone installation, we also note the need for continued research to understand the contextual factors that may explain such increases.
